# Hepatic Iron Overload following Liver Transplantation from a C282Y/H63D Compound Heterozygous Donor

**DOI:** 10.1155/2018/4298649

**Published:** 2018-05-31

**Authors:** E. Veitsman, E. Pras, O. Pappo, A. Arish, R. Eshkenazi, C. Feray, J. Calderaro, D. Azoulay, Z. Ben Ari

**Affiliations:** ^1^Liver Disease Center, Sheba Medical Center, Ramat Gan, Israel; ^2^Institute of Genetics, Sheba Medical Center, Ramat Gan, Israel; ^3^Sackler School of Medicine, Tel Aviv University, Tel Aviv, Israel; ^4^Department of Pathology, Sheba Medical Center, Ramat Gan, Israel; ^5^Hepato-Biliary-Pancreatic Surgery Department, Sheba Medical Center, Ramat Gan, Israel; ^6^Department of Hepatology, Henri Mondor Hospital, Créteil, France; ^7^Unite Inserm 955, France; ^8^Department of Pathology, Henri Mondor Hospital, Créteil, France; ^9^Department Hepato-Pancreato-Biliary Surgery and Liver Transplantation, Henri Mondor Hospital, Créteil, France

## Abstract

Hereditary hemochromatosis (HH) is a genetic disease associated with progressive iron overload, eventually leading in some cases to damage of parenchymal organs, such as the liver, pancreas, and heart. Although the gene had been identified (HFE), HH pathogenesis remains to be fully elucidated. We report here, for the first time, a case of inadvertent transplantation of a liver from a donor with C282Y/H63D compound heterozygosity into a nonhemochromatotic 19-year-old Caucasian male recipient with primary sclerosing cholangitis. Progressive iron overload occurred over 1.5 years, as observed in liver biopsies and iron studies, after ruling out secondary causes of iron overload. This case strengthens the hypothesis that the liver, rather than the small intestine, plays a primary role in the maintenance of iron homeostasis.

## 1. Introduction

Hereditary hemochromatosis (HH) is an autosomal recessive iron metabolism disorder, characterized by increased intestinal iron absorption and deposition in the parenchyma of liver, pancreas, heart, and other organs ([1], Pietrangelo A). HH remains the most commonly identified genetic disorder in Caucasians ([2], Bacon BR). C282Y homozygotes account for 80%-85% of HH patients. H63D and S65C mutations are also commonly detected but are generally only associated with iron overload in C282Y/H63D or C282Y/S65C compound heterozygotes ([2], Bacon BR). Recently mutations of other genes encoding iron regulatory proteins, such as hepcidin, hemojuvelin, transferrin receptor 2, and ferroportin, have been implicated in inherited iron overload syndromes [2].

There are four main pathophysiological mechanisms involved in iron overload: increased absorption of dietary iron in the upper intestine, decreased expression of iron regulatory hormone hepcidin, altered function of HFE, and iron-elicited tissue injury with fibrogenesis [2]. Yet, the exact pathophysiological mechanism of iron overload in HH remains elusive. In addition, the precise site of the main metabolic defect in HH patients has been a subject of controversy for many years. Duodenal cells, hepatocytes, and/or macrophages have been proposed to be targeted by HH.

The study in HFE-knockout mice reported by Vujic Spasic et al. [3] showed that conditional deletion of HFE in the liver induces a hemochromatosis phenotype, while its conditional deletion in duodenal cells or macrophages does not have the same effect on iron metabolism. Liver transplantation provides a unique opportunity to elucidate the role of the liver and the intestine in the pathogenesis of iron overload in HH ([4] **Dwyer JP)**. Here, we report the development of iron overload in a nonhemochromatotic patient following liver transplantation from a donor with compound C282Y/H63D heterozygosity.

## 2. Case Report

A 19-year-old Caucasian male presenting with severe primary sclerosing cholangitis underwent orthotopic liver transplantation and required a retransplant 5 weeks later due to a liver insufficiency caused by ligation of ruptured arterial pseudoaneurysm. He received more than 40 blood transfusions. The second donor was a 76-year-old male without a history of liver disease. The patient's postoperative course after retransplant included prolonged hemodialysis (8 weeks) due to acute kidney injury, cytomegalovirus (CMV) infection, hepatitis E infection, and hepatic artery stenosis in the anastomosis area, treated by angioplasty and stent insertion. Of note, hepatic artery stenosis resulted in ischemic-like cholangiopathy and prolonged cholestasis.

**Figure 1:**
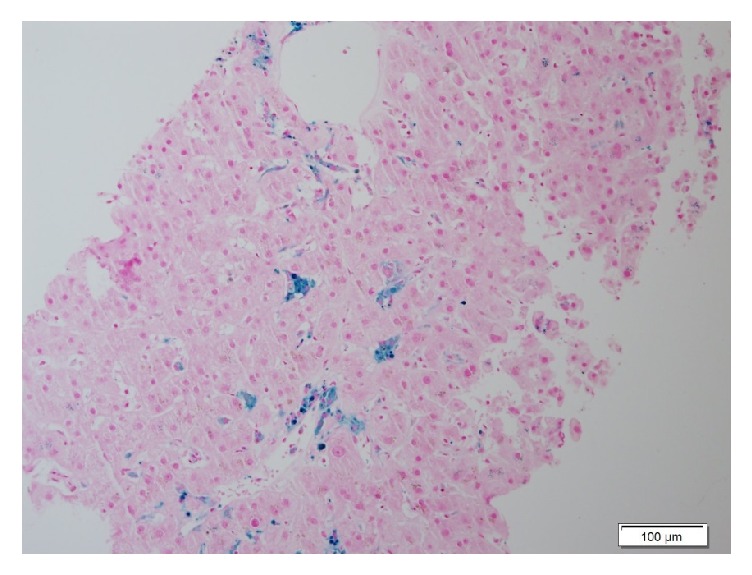
Liver biopsy 6 months after OLT showing hypertrophic macrophages containing iron and slight accumulation of hemosiderin in hepatocytes (Perls' stain).

The patient's condition stabilized eight months after transplantation. Cyclosporin and Myfortic were administered for immunosuppression, in addition to aspirin and ursodeoxycholic acid. A liver biopsy performed at that period revealed numerous hypertrophic, iron-loaded macrophages and severe bile duct damage and loss, consistent with early mild chronic rejection (Figure 1). Hemosiderosis was attributed to secondary iron overload, considering the numerous risk factors for this complication presenting before and after the retransplant (multiple blood transfusions, kidney injury, and CMV infection).

**Figure 2:**
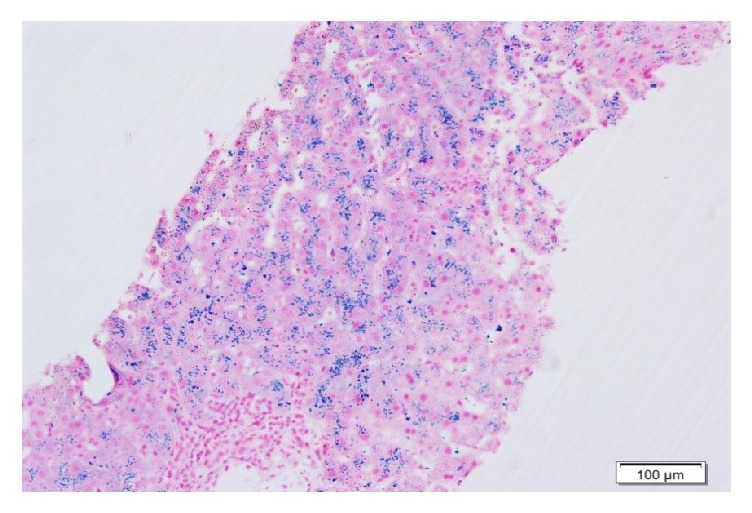
Liver biopsy 18 months after OLT demonstrating heavy granular iron deposition in hepatocytes and macrophages corresponding to hemosiderosis (Perls' stain).

Eight months later, elevation of liver enzymes was observed: alanine transaminase (ALT), 127 IU/L, aspartate transaminase (AST), 61 IU/L, alkaline phosphatase, 209 IU/L, and gamma-glutamyl-transpeptidase (GGT), 222 IU/ L. Extensive laboratory and radiologic evaluations showed no abnormalities, aside from iron-related parameters: serum iron, 110 ng/ml, ferritin, 3170 mg/dl (versus 29 mg/dL before transplant), transferrin, 119 mg/dL, and transferrin saturation, 66%. Repeated liver biopsy revealed sinusoidal fibrosis with mild cholangiolar proliferation. Iron staining showed significant accumulation of iron in macrophages and hepatocytes, consistent with marked hemosiderosis (Figure 2).

The combination of abnormal laboratory iron parameters and biopsy findings showing clear worsening of iron accumulation, without apparent new risk factors for secondary iron overload, led us to suspect primary rather than secondary hemosiderosis. Genetic testing of the patient's DNA ruled out preexisting HH and did not show any common HFE mutations (C282Y or H63D). Genetic high-resolution melt curve analysis of a biopsy sample revealed compound C282Y/H63D heterozygosity, confirming a genetic defect in the donor tissue, which elicited hereditary hemochromatosis in a recipient without any known HFE mutation.

Magnetic Resonance Imaging (MRI) performed or iron assessment revealed mild hepatic iron overload, consistent with 5 mg/gr, and did not show accumulation of iron in other organs: pancreas, adrenals, spleen, and heart.

Following the confirmation of the diagnosis, the patient was enrolled in a phlebotomy program.

## 3. Methods

Liver biopsies were fixed in 10% formalin, embedded in paraffin, and stained with hematoxylin-eosin, trichrome, reticulin, periodic acid Schiff (PAS), and iron stains, according to standard methods.

### 3.1. Molecular Analyses

The C282Y and H63D mutations were detected by Sanger sequencing using the primers: F- ACACAGCTGATGGTATGAGTTGAT and R-ATGAAAAGATGAAAAGCTCTGACAA; F-AGAAGGAAGTGAAAGTTCCAGTCTT and R-ATCTCACTGCCATAATTACCTCCTC, respectively. Amplification was carried out in a 25 *µ*L reaction containing 50 ng DNA, 10 ng of each primer, and 12.5 *µ*L RM (Thermo Scientific). After an initial denaturation of 2 min at 95°C, 30 cycles were performed (95°C, 60°C and 72°C, for 30 sec each), followed by a final extension of 10 min at 72°C and sequencing, using an automated ABI Prism 3100 Genetic Analyzer (Perkin Elmer).

## 4. Discussion

Here, we present a first report of inadvertent transplantation of a liver from a donor with C282Y/H63D compound heterozygosity into a nonhemochromatotic recipient. The number of cases shed light onto current knowledge (Table 1) of HH pathogenesis, which remains to be fully elucidated.

The possible role of the liver versus the intestine in the pathogenesis of HH has been the subject of long existing controversy. The “tale of two sites” began long before the development of HFE genetic studies and liver transplantation has become the greatest contributor in the understanding of HH pathophysiology. In the early 1990s, evidence ruling out an exclusively intrahepatic defect began to accumulate. Adams et al. [8] described a biopsy-proven rapid decline of hepatic iron levels in a hemochromatosis liver, which was inadvertently transplanted into a recipient suffering from acute liver failure. A few years later, when genetic analyses became available, the same team presented an even more informative case when both the liver and the intestine from a C282Y homozygous patient were transplanted into a normal recipient [5]. Twenty-one months later, the recipient developed the biochemical abnormalities typical of early hemochromatosis (increased transferrin saturation with still normal hepatic iron concentration and normal serum ferritin). The authors concluded that both cases supported their hypothesis regarding existence of a site-specific fundamental defect in the intestine of hemochromatosis patients [5, 8]. Further support of this theory was provided by several case reports of non-HH recipients who received livers from HH donors [9] and development of a decrease in iron overload after transplant in the majority of these patients.

Strong arguments against this hypothesis were presented in early 2000s, with claims that if HH is due to an intestinal defect, liver transplantation would not cure the hemochromatosis and iron would be expected to reaccumulate [10]. Yet, a number of studies failed to observe any long-term (up to 12 years) reaccumulation of iron after liver transplantation for HH [11–13]. In 2003, Wigg AJ et al. [6] described, for the first time, the development of phenotypic hereditary hemochromatosis in a non-HH liver transplant recipient following transplantation of a liver from a C282Y heterozygous donor. In their case, a novel pathogenic missense mutation of the HFE gene, R6S, was discovered in the recipient. The authors hypothesized that an interaction between R6S heterozygosity in the recipient and C282Y heterozygosity in the donor liver drove development of iron overload in the patient. In essence, the authors suggested that a hepatic defect is required for expression of HH and that the intestinal HFE genotype can impact but is not the exclusive determinant of iron metabolism [6].

Since then, a growing body of evidence has supported the hypothesis that the liver, rather than the intestine, plays a primary role in the maintenance of iron homeostasis. Ismail MK et al. [7] presented another case of posttransplant iron overload following transplantation of a C282Y homozygous liver into an H63D heterozygous recipient. The authors suggested involvement of hepcidin, a relatively newly discovered iron flow regulator [14, 15]. They also speculated that heterozygosity for the H63D mutation in the presence of a liver with the C282Y mutation may be much more consequential, as in the case of Wigg et al. [6].

Finally, Dwyer JP et al. [4] reported a case of inadvertent transplantation of a liver with C282Y homozygosity from an HH donor into a C282Y wild type (no hemochromatosis and no HFE mutations) recipient with fulminant hepatic failure due to hepatitis B reactivation. The development of clinically significant hemochromatosis was detected in a recipient two years following the transplant. In this case, only C282Y homozygosity (without any mutation in a recipient) was sufficient to trigger hemochromatosis in the recipient. In the present case, several risk factors for secondary iron overload presented immediately after transplantation (multiple blood transfusions and hemodialysis), but no additional risk factors accumulated between the first and the second biopsy. Thus, transfer of the genetic defect of hemochromatosis from the donor liver to the recipient is the most probable explanation for iron overload development with features of primary hemochromatosis. We hypothesize that C282Y/H63D heterozygosity in the donor liver triggered development of the HH phenotype in the recipient who did not have any evidence of pathogenic HFE mutations. This case provides support for the hypothesis that the liver plays the most critical role in iron homeostasis.

Our case study has a few limitations. First, hepcidin, as a well-established and key regulator of iron flow from duodenal hepatocytes to the liver, was not evaluated in our patient. The use of hepcidin assays is limited for research purposes only and is still not available in any medical institution in Israel. Second, the occurrence of clinical iron overload in compound C282Y/H63D heterozygosity according to the recent data is extremely rare [16]. The concomitant existence of an additional unknown genetic defect in the recipient (coding the HH genes or other genes involved in iron metabolism), which possibly triggered iron overload, was considered but was not evaluated. In addition, there is a possibility of contribution of nongenetic factors such as the multiple transfusions and renal dialysis.

In conclusion, we presented the first report of inadvertent transplantation of a liver from a donor with C282Y/H63D compound heterozygosity into a nonhemochromatotic recipient. The case strengthens the hypothesis that the liver, rather than the small intestine, plays a primary role in the maintenance of iron homeostasis.

## Figures and Tables

**Table 1:** 

Reference #	Year of publication	Donor	Recipient	Reason for transplant	Time from OLT to HH development (months)	Outcome
[5]	1999	C282Y homozygote liver & intestine	Non-HH	Cholestatic liver disease & short bowel syndrome	21	Biochemical abnormalities consistent with HH

[6]	2003	C282Y heterozygote liver	new missense mutation R6S	Alcoholic cirrhosis	49	Treated with phlebotomy

[7]	2009	C282Y homozygote liver	H63D heterozygote	HBV+HCV +ethanol	60	Died due to lung cancer

[4]	2011	C282Y homozygote liver	Non-HH	Fulminant HBV reactivation	24	Treated with phlebotomy

Present case	2016	C282Y/H63D compound heterozygote	Non-HH	PSC and Crohn's disease	18	Planned for phlebotomy
